# Robust innate immune responses at the placenta during early gestation may limit *in utero* HIV transmission

**DOI:** 10.1371/journal.ppat.1009860

**Published:** 2021-08-25

**Authors:** Erica L. Johnson, Dominika Swieboda, Amanda Olivier, Elizabeth Ann L. Enninga, Rana Chakraborty

**Affiliations:** 1 Department of Microbiology, Biochemistry, and Immunology, Morehouse School of Medicine, Atlanta, Georgia, United States of America; 2 Department of Pediatrics, Division of Infectious Diseases, Emory University School of Medicine, Atlanta, Georgia, United States of America; 3 Department of Pediatric and Adolescent Medicine, Mayo Clinic College of Medicine and Science, Rochester, Minnesota, United States of America; University of Wisconsin, UNITED STATES

## Abstract

In 2019, >90% of new HIV infections in infants globally occurred vertically. Studies suggest intrauterine transmission most often occurs in the third trimester; however, there are no mechanistic studies to support these observations. We therefore obtained early/mid-gestation and term placentae from 20 HIV/Hepatitis B/CMV negative women. Isolated primary placental macrophages (Hofbauer cells [HCs]) were exposed to HIV-1_BaL_ and/or interferon (IFN)-α, IFN-β, IFN-λ1, and RIG-I-like receptor (RLR) agonists. qRT-PCR, FACS, ELISA, Luminex, and Western blot analyses determined expression of activation markers, co-receptors, viral antigen, cytokines, antiviral genes, and host proteins. Early gestation HCs express higher levels of CCR5 and exhibit a more activated phenotype. Despite downregulation of CCR5, term HCs were more susceptible to HIV replication. Early gestation HCs displayed a more activated phenotype than term HCs and HIV exposure lead to the further up-regulation of T-cell co-stimulatory and MHC molecules. Limited HIV replication in early/mid gestation HCs was associated with increased secretion of anti-inflammatory cytokines, chemokines, and a more robust antiviral immune response. In contrast, term HCs were more susceptible to HIV replication, associated with dampening of IFN-induced STAT1 and STAT2 protein activation. Treatment of early/mid gestation and term HCs, with type I IFNs or RLR agonists reduced HIV replication, underscoring the importance of IFN and RLR signaling in inducing an antiviral state. Viral recognition and antiviral immunity in early gestation HCs may prevent *in utero* HIV infection, whereas diminished antiviral responses at term can facilitate transmission. Defining mechanisms and specific timing of vertical transmission are critical for the development of specific vaccines and antiviral therapeutics to prevent new HIV infections in children globally.

## Author summary

Mother-to-child transmission is the main source of HIV infection in children globally. Studies suggest vertical transmission most often occurs late in the third trimester; however, there are no studies to support these observations. Our study shows that gestational age plays a significant role in the ability of placental macrophages to generate robust antiviral responses, which are necessary to prevent or reduce viral burden. Specifically, we show that viral recognition by RIG-I-like receptors and robust antiviral immune responses in placental cells during early gestation may prevent *in utero* HIV infection. We also demonstrate that term placental macrophages are limited in their antiviral capacity due to restricted type I IFN signaling. Understanding the mechanisms and timing of vertical transmission are important to understand for the development of specific vaccines and antiviral therapeutics to prevent new HIV infections in children globally.

## Introduction

In 2018, an estimated 160,000 new pediatric HIV infections occurred worldwide, most by mother-to-child (or vertical) transmission (MTCT). Even with optimal adherence, maternal antiretroviral therapy (ART) reduces but does not eliminate MTCT of HIV [1–4], which can occur *in utero*, intrapartum, and postpartum through breastfeeding. We have shown that a distinct immunological repertoire in placental macrophages (Hofbauer cells [HCs]), exhibiting remarkable plasticity, may be temporally regulated during gestation [5–8]. These phenotypic alterations may influence HIV intrauterine transmission to the fetus/neonate, which is thought to mainly occur during the third trimester [9, 10] based on low rates of viral detection by nucleic acid testing on fetal tissue from abortions in the first and second trimester [11], as well as by statistical modeling [12]. An initial case report from 1986 documented detection of HIV antigens in a fetus at 15 weeks’ gestation [13]. Subsequent reports in 1991 and 1995 provided compelling evidence for late intrauterine HIV transmission using PCR [12, 14]. Statistical models trended (*P* = .061) towards decreased frequency of early *in utero* HIV infection among infants born to women with HIV-infection [15]. In addition, the Perinatal HIV Prevention Trial (PHPT-I) from Thailand in 2000 documented an *in-utero* transmission rate of 5.1% when zidovudine was initiated at 35 weeks versus 1.6% when initiated at 28 weeks [16], suggesting that most intrauterine HIV transmission events occurred between 28 to 36 weeks gestation [17]. Despite these reports and associations over 30 years, there are a lack of basic mechanistic studies to explain why transmission tends to occur near the end of gestation [18, 19].

The placenta is immunologically active and unique in its ability to sustain a healthy pregnancy and offset infection [20–22]. This organ also remains a target following maternal infection with rubella, CMV, HSV, HIV, Zika, or hepatitis B and C virus [23]. During maternal HIV infection, virions can interact with placental macrophages (Hofbauer cells [HCs]) after breaching the trophoblast layer prior to entering the fetal circulation [24–26]. HCs express HIV (co) -receptors CD4, CCR5, CXCR4, and DC-SIGN on their cell surface [25, 27–29]. Since these cells are uniquely positioned between maternal and fetal circulations, HCs are recognized as key mediators for MTCT of HIV [25, 27–29]. Several studies show that HIV can be detected in placentae from both transmitting and non-transmitting women, and sequester virus *in vivo* during early and late gestation [30–35]. Placental cells also express pattern-recognition receptors (PRRs) [22, 36–41]. Viral proteins activate PRRs, which in turn stimulate transcription factor NF-kB and/or type I IFN. Type I IFN, through the IFN-α/β receptor (IFNAR), induce a multitude of IFN-stimulated genes (ISGs) that can block viral replication and alert the immune system in response to virus [33, 42, 43]. Along with type I IFNs, type III IFNs or IFN-λ regulate a similar set of genes to restrict viral infection [44, 45]. However, type I IFNs act systemically, while IFN-λ primarily targets mucosal barriers (i.e. the placenta), circumventing significant inflammatory risk associated with type I IFN responses. We previously demonstrated that viral PRRs and type I/III IFN responses in HCs may be temporally regulated and that phenotypic and functional changes of HCs throughout gestation may be responsible for the differential effects and outcomes of maternal infection during pregnancy [5].

In this study, primary HCs isolated from early/mid-gestation placental tissue and term were exposed to HIV-1_BaL_ and/or IFN-α, IFN-β, IFN-λ, and RLR agonists. We determined expression of activation markers, co-receptors, viral antigen, cytokines, antiviral genes, and host proteins. We showed that term HCs were more susceptible to HIV replication and exhibited increased HIV gene expression compared to early gestation HCs. Early gestation HCs displayed a more activated phenotype than term HCs and HIV exposure lead to further up-regulation of T-cell co-stimulatory and MHC molecules. Limited HIV infection and replication in early/mid gestation HCs correlated with increased secretion of anti-inflammatory cytokines and a more robust antiviral immune response compared to the dampened antiviral response shown by term HCs. In addition, treatment of early/mid gestation and term HCs, with type I IFNs or RLR agonists completely blocked or significantly reduced HIV replication, emphasizing the importance of the these signaling pathways in inducing an antiviral state at the placenta during viral infection. Our findings also suggest that dampening of STAT1 and STAT2 protein activation in term HCs may account for observed increases in HIV replication. Interestingly, unlike early gestation HCs, HIV-1 infection of term cells induced significant upregulation of STAT5 phosphorylation, which alludes to the unique ability of this STAT protein to promote HIV infection in macrophages [46]. *In sum*, we show that viral recognition and robust antiviral immune responses in placental cells during early gestation may prevent *in utero* HIV infection and that diminished antiviral responses observed in term HCs may promote vertical transmission.

## Results

### Early/mid-gestation HCs express higher protein surface levels of CCR5 compared to term HCs

**Fig 1:**
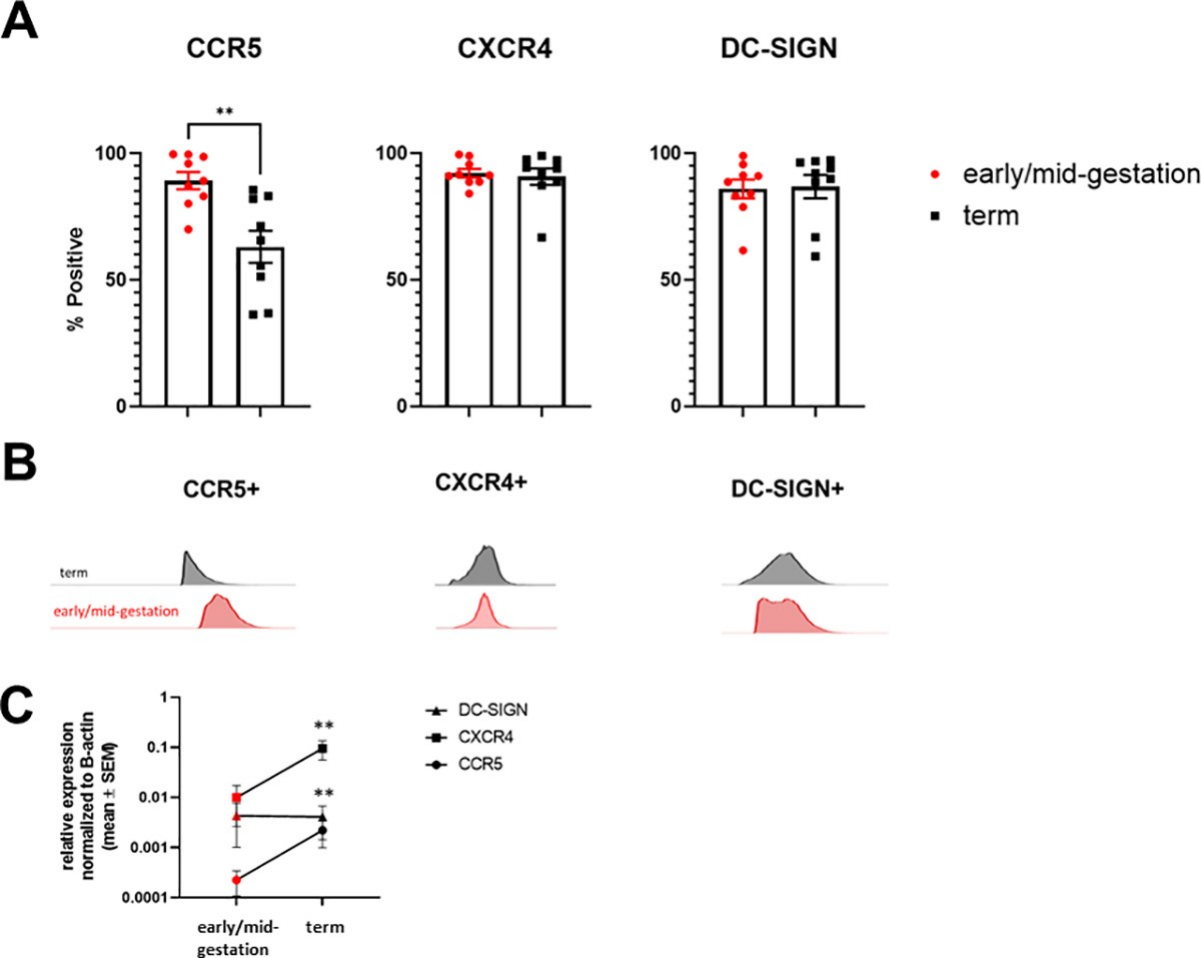
Early/Mid-Gestation HCs express Higher Protein Surface Levels of CCR5 compared to Term HCs. *A*, Surface expression of CCR5, CXCR4 and DC-SIGN was determined by flow cytometry on freshly isolated HCs from early/mid-gestation (red markers) and term (black markers) placental tissue. Data shown are expressed as the mean ± standard error of biological triplicates from 10 individual donors analyzed by unpaired t-test analysis using the two-stage linear step-up procedure of Benjamini, Krieger and Yekutieli. **p < 0.001 indicates significance between early/mid-gestation HCs compared to term HCs. *B*, Representative flow cytometry histograms of typical distributions of fluorescence intensity (along x axis) are provided. *C*, Messenger RNA levels were measured by qRT-PCR to determine the relative expression of CCR5, CXCR4, and DC-SIGN. Gene expression data are represented as fold change normalized to β-actin (ΔΔ cycle threshold method). **p < .001 indicate significance between early/mid-gestation HCs compared and term HCs.

To understand whether gestational age impacts HIV susceptibility in HCs, we measured expression of the HIV co-receptors CCR5, CXCR4 and DC-SIGN in HCs isolated from early/mid-gestation (12–24 week [wk] gestational age) and term (>37 wk) placentae. Analysis by flow cytometry revealed a higher frequency of CCR5^+^ HCs in early/mid gestation (p = 0.0024). An average of 89.3% of HCs isolated from placentae between 12-22-weeks’ gestation were CCR5^+^, compared to 63.1% at term. Early/mid gestation and term CXCR4^+^ and DC-SIGN^+^ cells were constant in frequency, suggesting their expression is not dependent on gestational age (Fig 1A and 1B). Analysis by quantitative reverse transcription-PCR (qRT-PCR) showed a significant increase in CCR5 (p<0.001) and CXCR4 (p<0.001) mRNA expression in term HCs, compared to early/mid-gestation (Fig 1C). Similar to surface protein expression, DC-SIGN mRNA expression remained constant across gestation. These data initially suggested to us that HCs may be more susceptible to HIV infection in early/mid-gestation, compared to term.

### Paradoxically, term HCs are more permissive to HIV infection than early/mid-gestation HCs

**Fig 2:**
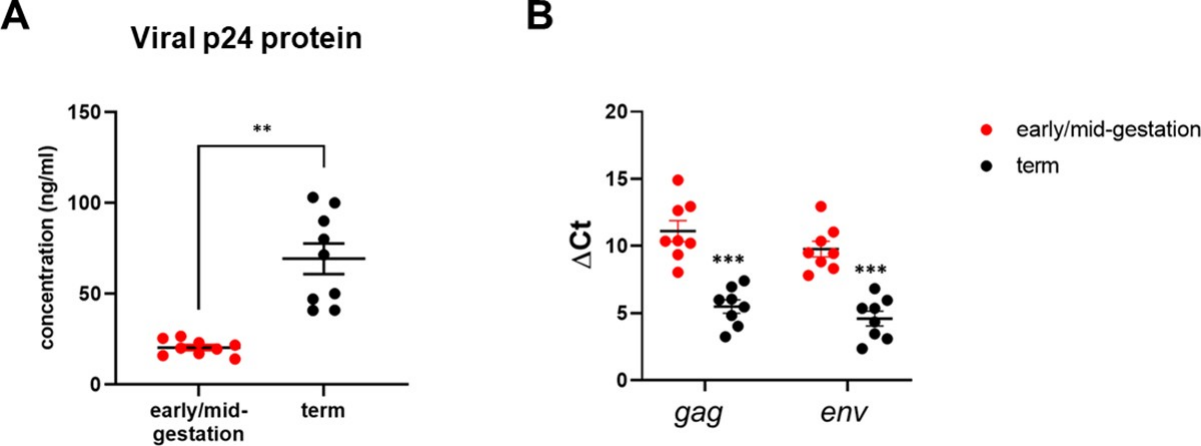
Term HCs are more Permissive to HIV Infection than Early/Mid-Gestation HCs. *A*, Human placental macrophages (HCs) were isolated from freshly obtained placenta from donors at term (black markers) or during early/mid-gestation (red markers). HC were infected with HIV BaL strain (HIV-1_BaL_) at a multiplicity of infection of 0.1. HIV-1 replication was measured in the cell supernatants at 5 days post infection by HIV-1 p24 viral antigen enzyme-linked immunosorbent assay. Data shown represent individual donors (n = 9) analyzed by unpaired t-test analysis using the two-stage linear step-up procedure of Benjamini, Krieger and Yekutieli. **p < 0.001 indicates significance between early/mid-gestation HCs compared to term HCs. *B*, Six days after viral infection, HIV-infected HC’s messenger RNA levels were measured by qRT-PCR to determine the relative expression of gag and env. Gene expression data are represented as ΔCt (normalized to β-actin). Data shown represent individual donors (n = 9) analyzed by unpaired t-test analysis using the two-stage linear step-up procedure of Benjamini, Krieger and Yekutieli. *** p< 0.0001 indicate significance between early/mid-gestation HCs compared and term HCs.

Several studies suggest that *in utero* transmission of HIV via the placenta likely occurs during the third trimester [9, 10]; however, there are a lack of mechanistic studies to explain this observation. To determine whether gestation impacts viral replication in placental target cells, we compared replication of HIV_BaL_ in HCs isolated from early/mid-gestation (12–24 wk gestational age) and term (>37 wk) placenta. p24 levels were detected in supernatant 5-days post infection (dpi). Surprisingly, mean production of p24 antigen in term HCs infected with HIV was significantly increased (p = 0.004), compared with production in early/mid-gestation HCs (Fig 2A). In addition, we measured HIV viral gene transcription 5 days after infection, using qRT-PCR. We noted that term HCs infected with HIV exhibited lower median cycle threshold (Ct) values (± standard error [SE]) for *gag* (5.49 ± 1.42) and *env* (4.59 ± 1.54) transcripts than that of infected early/mid-gestation HCs *gag* [11.1 ± 2.22] and *env* [9.78 ± 1.65]. Significantly lower *gag* and *env* Ct values in term HCs indicates higher levels of viral transcription, compared with early/mid-gestation cells. These results demonstrate that term HCs are more susceptible to HIV infection and more replication competent, compared to early/mid-gestation HCs. Our data support previous clinical observations that *in utero* transmission likely occurs in the third trimester.

### HIV infection induces activation of HCs

**Fig 3:**
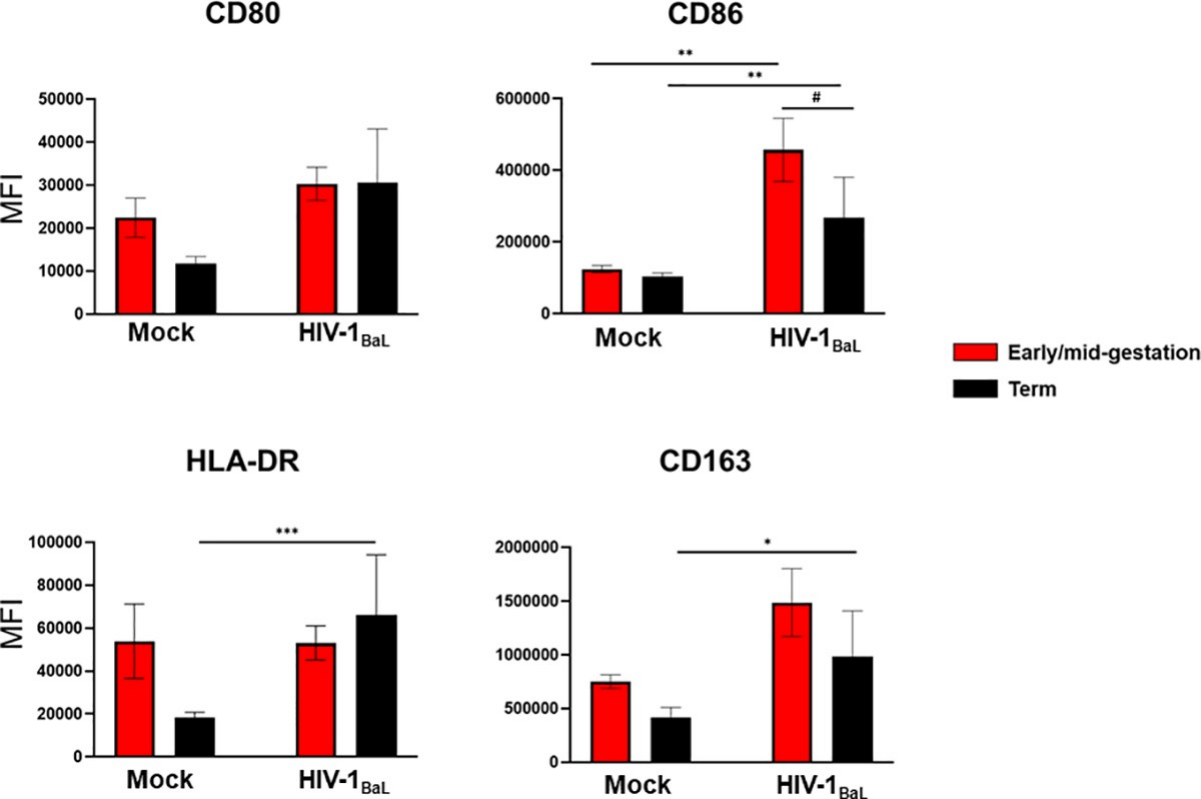
HIV Infection Induces Activation of HCs. Hofbauer cells from early gestation (red bars) and term (black bars) placentae were infected with HIV-1_BaL_ or mock infected. Surface-marker expression of CD80, CD86, HLA-DR, and CD163 was determined by flow cytometry 48 hours post-infection (hpi). Data shown are expressed as the mean ± standard error of biological triplicates from 10 individual donors analyzed by paired t-test analysis (mock infected HCs vs HIV infected HCs) and unpaired t-test analysis (early/mid-gestation HCs vs. term HCs)using the two-stage linear step-up procedure of Benjamini, Krieger and Yekutieli. *** p< 0.0001, **p < .001, and *p < .05 indicate significance between HIV-infected HCs and mock-infected HCs. #p < .05 indicate significance between early/mid-gestation HCs and term HCs.

Macrophages can respond to their microenvironment by altering their activation phenotype resulting in the broad classification of classical (M1) or alternative (M2) macrophage activation. Common surface markers to identify macrophage activation include CD80, CD86 and the class II major histocompatibility complex (MHC) molecule HLA-DR for M1 and CD163 for M2 [47]. Recently, our group documented that early/mid-gestation is associated with an abundance of activated HCs, whereas term cells appear less galvanized [5]. To investigate whether HIV infection differentially activates placental macrophages throughout gestation, we measured cell surface expression of the CD80, CDC86, HLA-DR, and CD163, at 48hpi with HIV_BaL_ (Fig 3). Following infection, we observed significant activation of HCs across all gestational ages. HIV-infected HCs isolated at term upregulated surface protein expression of CD86, HLA-DR, and CD163, while early/mid-gestation HIV-infected HCs showed significant increases in the surface expression of CD86. Although early/mid-gestation HCs exhibit a more activated phenotype than term HCs at a basal level, upon HIV infection term HCs become more galvanized. This increase in activation status correlates with enhanced viral replication in term HCs. These data suggest that HIV has the potential to prime and activate HCs upon infection.

### HIV infection of early/mid-gestation HCs induces the secretion of IL-6 and IL-10

**Fig 4:**
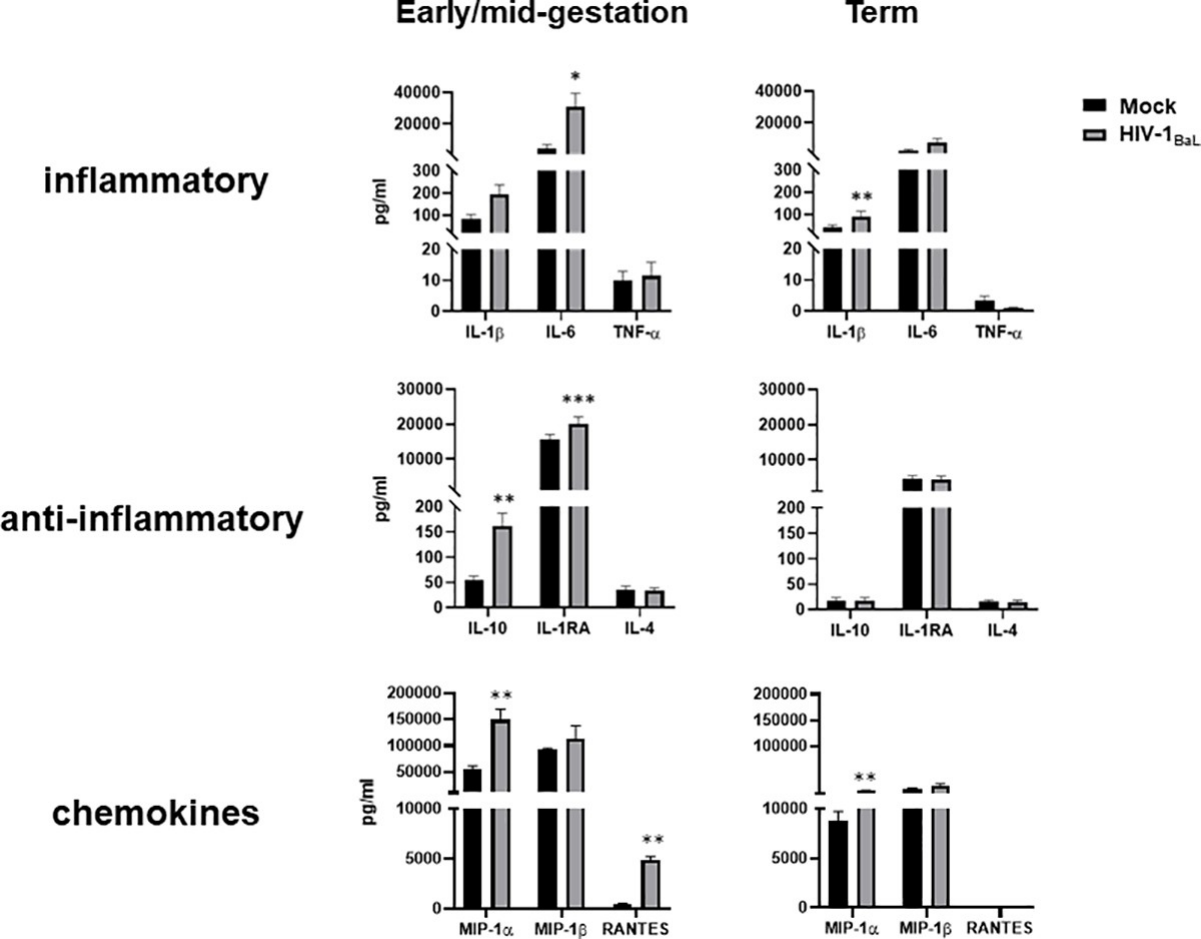
HIV Infection of Early/Mid-Gestation HCs Induces the Secretion of IL-6 and IL-10. *A*, Hofbauer cells (isolated from early/mid-gestation and term) were infected with HIV-1BaL (black bars) or mock-infected (gray bars). Quantification of inflammatory cytokine, anti-inflammatory cytokine, and chemokine protein levels were determined in the supernatants by Luminex following 48 hours of *in vitro* culture. All values are represented as pg/mL. Data shown are expressed as the mean ± standard error of biological triplicates from 10 individual donors analyzed by paired t-test analysis using the two-stage linear step-up procedure of Benjamini, Krieger and Yekutieli. *** p< 0.0001, **p < .001, and *p < .05 indicate significance between HIV-infected HCs and mock-infected HCs.

During pregnancy, Th_2_ (e.g., IL-10) and Th_3_ responses (e.g., transforming growth factor β), which support pregnancy, are enhanced, whereas Th_1_ cytokines (i.e., IL-12, and IFN-γ), which are potentially detrimental to the foreign fetus, are suppressed [6, 48]. Maternal infections in pregnancy can trigger pro-inflammatory responses leading to intrauterine infection [49], via activation of PRRs with increased secretion of T_h_1 cytokines, including IL-1 and TNF-α [50]. These contribute to poor pregnancy outcomes including disruption to fetal immunity [51, 52]. Here we assessed cytokine and chemokine release following HIV infection of HCs isolated early/mid-gestation and at term (Fig 4). Despite increases in cellular activation, term HCs infected with HIV failed to induce substantial increases in cytokine and chemokine secretion, compared to paired mock-infected cells. In addition, term cells at basal levels express significantly lower concentrations of immunoregulatory cytokines (IL-10 and IL-1RA) and β-chemokines (MIP1α and MIP1β), compared to early/mid-gestation HCs. In contrast, HIV infection of early/mid-gestation HCs prompted robust secretion of IL-6 and IL-10. HIV infection also induced significant upregulation of MIP1α and RANTES in early/mid-gestation HCs. Together, our data suggest that term HCs are compromised in their ability to promote anti-inflammatory responses upon exposure to HIV.

### HIV induces type I IFN production in early/mid-gestation HCs

**Fig 5:**
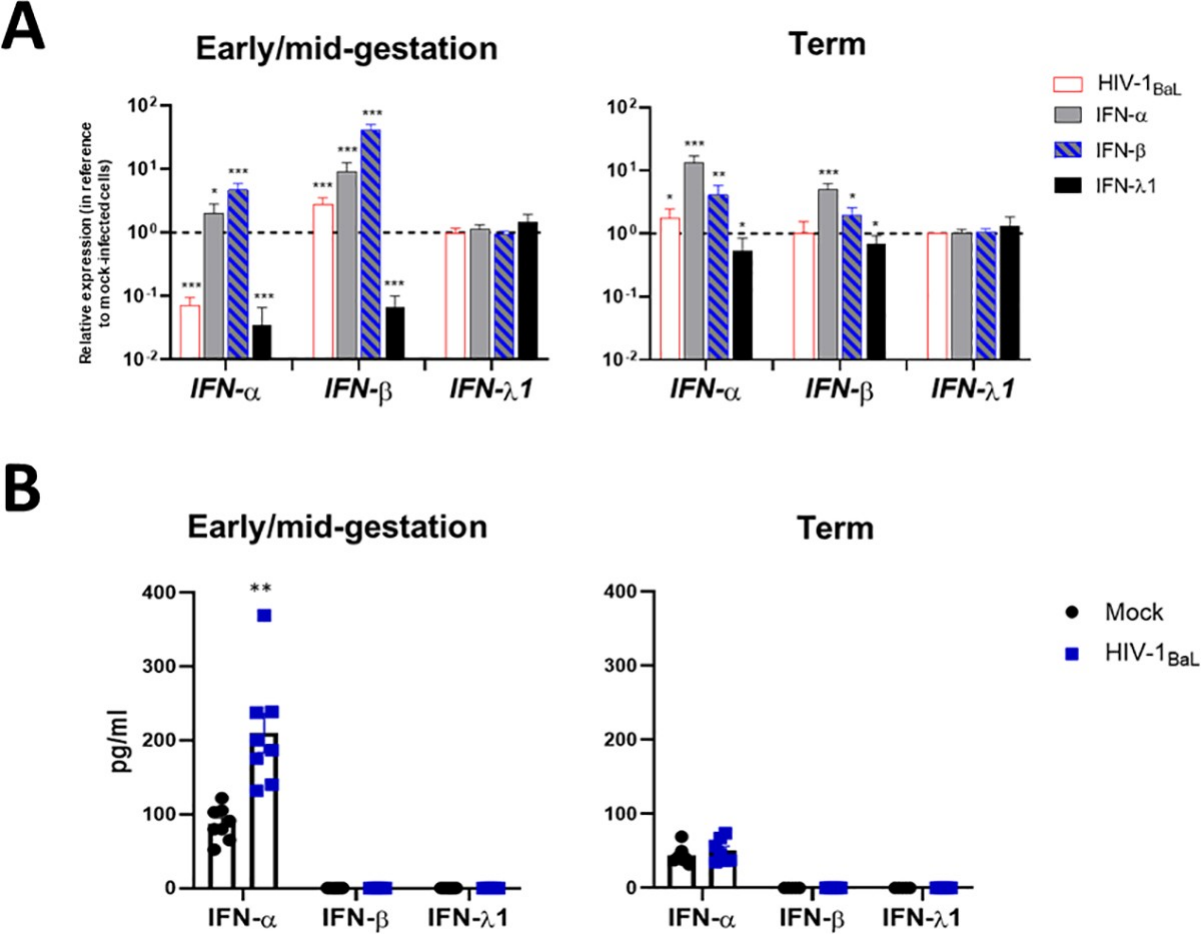
HIV Induces Type I IFN Production in Early/Mid-Gestation HCs. Hofbauer cells (isolated from early/mid-gestation and term) were infected with HIV-1_BaL_ and treated with IFN-α (100 IU/mL), IFN-β (100 ng/mL), IFN-λ1 (100 ng/mL), RIG-I agonist (10ng/10,000 cells) or mock-infected. *A*, Messenger RNA levels of the type I IFNs (IFN-α and IFN-β) and the type III IFN (IFN-λ1) was measured by qRT-PCR. Gene expression data are represented as fold change relative to time-matched, mock-infected cells (gene expression normalized to β-actin–ΔΔ cycle threshold method). Data shown are expressed as the mean ± standard error of biological triplicates from 10 individual donors analyzed by paired t-test analysis using the two-stage linear step-up procedure of Benjamini, Krieger and Yekutieli. *** p< 0.0001, **p < .001, and *p < .05 indicate significance between mock-infected and HIV-infected or IFN-treated cells. *B*, Quantification of IFN-α, IFN-β, and IFN-λ1 protein levels were determined in the supernatants by Luminex following 48 hours of *in vitro* culture. All values are represented as pg/mL. Data shown are expressed as the mean ± standard error of biological triplicates from 10 individual donors analyzed by paired t-test analysis using the two-stage linear step-up procedure of Benjamini, Krieger and Yekutieli. **p < .001 indicate significance between HIV-infected HCs and mock-infected HCs.

During viral infection, early innate immune signaling triggers the production of type I and type III IFNs, and antiviral effector molecules that block viral replication [53]. In addition, viral sensing by PRRs is a key step in responding to viral infections. To determine impact of gestation on whether placental macrophages trigger IFN responses during HIV infection, we measured mRNA concentrations of the type I IFNs (IFNα and IFNβ) and type III IFN (IFNλ1). We also measured the secretion of IFNα, IFNβ and IFNλ1 proteins in HC supernatant following infection by HIV at 48hpi. At the mRNA level, HIV-infection of early/mid-gestation HCs induced an approximate 10-fold decrease in the mRNA expression of IFNα, while IFN-β transcription was significantly upregulated (Fig 5A). In contrast, HIV infection of term cells only upregulated the mRNA expression of IFN-α. As expected, treatment with the type I IFNs, induced their corresponding mRNA transcription in both cellular subsets. However, we did not detect changes in IFNλ1 mRNA expression across gestational ages with or without infection or treatments. Similarly, at the protein level, HCs across gestation and with or without HIV-infection failed to secrete IFNλ1 (Fig 5B). However, treatment with the IFNλ1 demonstrated a block in the transcription of the IFN-α and IFN-β in early/mid-gestation HCs. At the protein level, all HCs also failed to secrete IFN-β, despite significant increases at the level of transcription (Fig 5B). In contrast, both subsets of HCs expressed IFNα at a basal level. Specifically, early/mid-gestation HCs produced more IFNα at a basal level, compared to term HCs. In addition, HIV infection of these cells significantly increased IFNα production, while HIV infection of term HCs did not impact IFN-α secretion. These findings suggest that the production of IFN-α upon HIV infection may play a key role in protection at the placenta, particularly in early/mid-gestation HCs.

### HIV-1 infection induces an antiviral state within early/mid-gestation HCs

**Fig 6:**
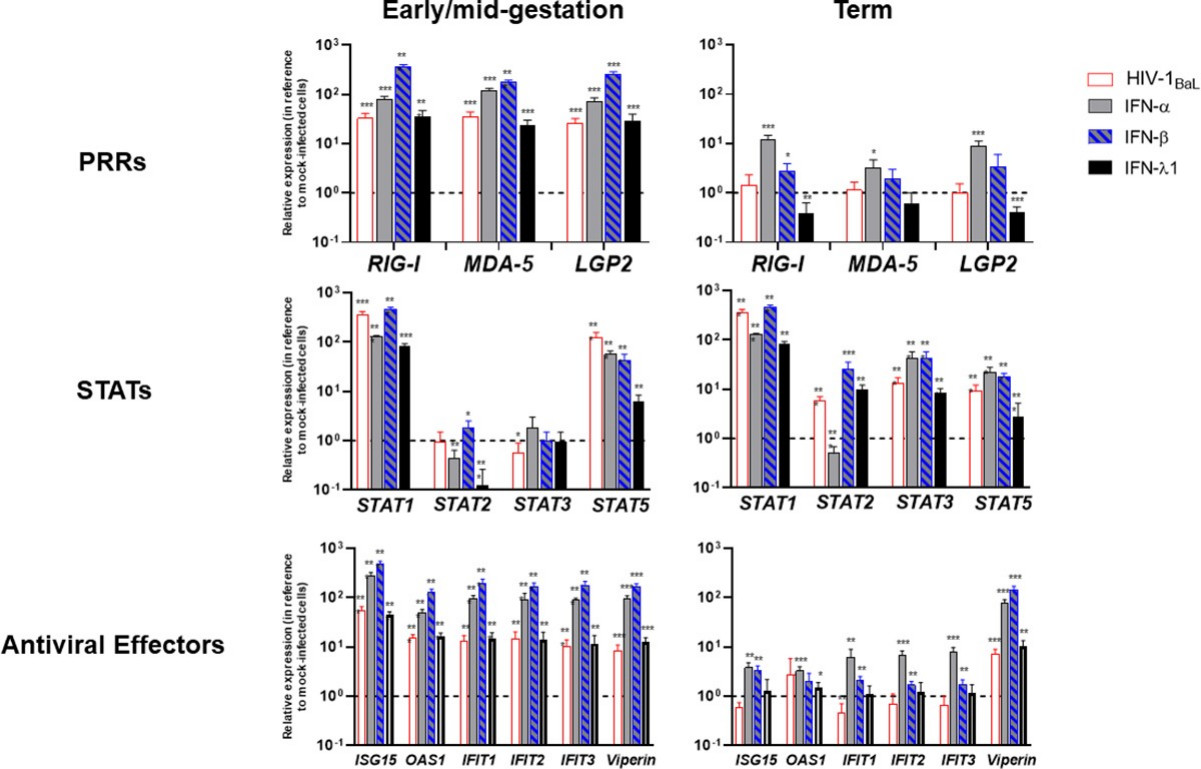
HIV-1 Infection Induces an Antiviral State within Early/Mid-Gestation HCs. *A*, Hofbauer cells (isolated from early/mid-gestation and term) were infected with HIV-1_BaL_, mock-infected or treated with IFN-α (100 IU/mL), IFN-β (100 ng/mL) or IFN-λ1 (100 ng/mL). The type I IFN response was evaluated by measuring messenger RNA levels of the Pattern Recognition Receptors (PRRs; RIG-I, MDA-5, and LGP2), signal transducer and activator of transcription (STAT1, -2, -3, and -5), along with key antiviral effectors (ISG15, OAS1, IFIT1, IFIT2, IFIT3, and Viperin) by qRT-PCR. Gene expression data are represented as fold change relative to time-matched, untreated cells (gene expression normalized to β-actin–ΔΔ cycle threshold method). Data shown are expressed as the mean ± standard error of biological triplicates from 10 individual donors analyzed by paired t-test analysis using the two-stage linear step-up procedure of Benjamini, Krieger and Yekutieli. *** p< 0.0001, **p < .001, and *p < .05 indicate significance between mock-infected and HIV-infected or IFN-treated cells.

To characterize the type I and type III IFN response in placental HCs throughout pregnancy and following HIV infection, we measured mRNA concentrations of the RLRs (RIG-I, MDA-5, and LGP2) and key host antiviral effectors, including the signal transducer and activator of transcription (STAT) proteins by qRT-PCR. Comparisons were made between mock-infected cells vs. cells treated with IFNα (1000 U/ml), IFNβ (1000 U/ml) or IFNλ1 (100 ng/ml) or infected with HIV-1_BaL_ for 24 hours. HIV infection of early/mid-gestation HCs generated an overall robust type I IFN response, characterized by significant upregulation of the RLRs, STAT1, STAT5, and all the antiviral effectors tested (ISG15, OAS1, IFIT1, IFIT2, IFIT3, and Viperin) (Fig 6). In comparison, HIV infection of term HCs minimally triggered IFN signaling. There were no significant differences in RLR expression between mock and HIV-infected term HCs. In addition, Viperin was the only antiviral effector that displayed substantial upregulation in term HCs, in response to HIV. However, HIV infection significantly impacted the mRNA expression of the STAT proteins. STAT1, STAT2, STAT3, and STAT5 transcription was significantly upregulated following HIV infection of term HCs. The increase observed by STAT2 and STAT3 was significantly greater than the production by early/mid-gestation HCs (Fig 6).

At a basal level, early/mid-gestation HCs exhibit a more profound antiviral state when compared to term [5]. As expected, treatment with the type I IFNs upregulated the expression of RLRs, STATs, and the antiviral effectors in HCs isolated at different timepoints throughout pregnancy. However, the early/mid-gestation HCs generated a more robust antiviral response to the type I IFNs, compared to term cells. RLR expression following type I IFN treatment was 10- to 100-fold greater in early/mid-gestation cells, compare to term. In addition, mRNA expression of ISG15, OAS1, IFIT1, IFIT2, and IFIT3 was significantly higher in type I IFN-treated early/mid-gestation HCs compared to term. Interestingly, the type III IFN, IFNλ1, mounted a robust RLR and IFN response in early/mid-gestation HCs comparable to the cells treated with IFN-α and IFN-β. However, this response was dampened in HCs isolated at term. Taken together, gestational age plays a significant role in the ability of HCs to generate robust antiviral responses.

### HIV infection is blocked by type I and type III signaling across gestation

**Fig 7:**
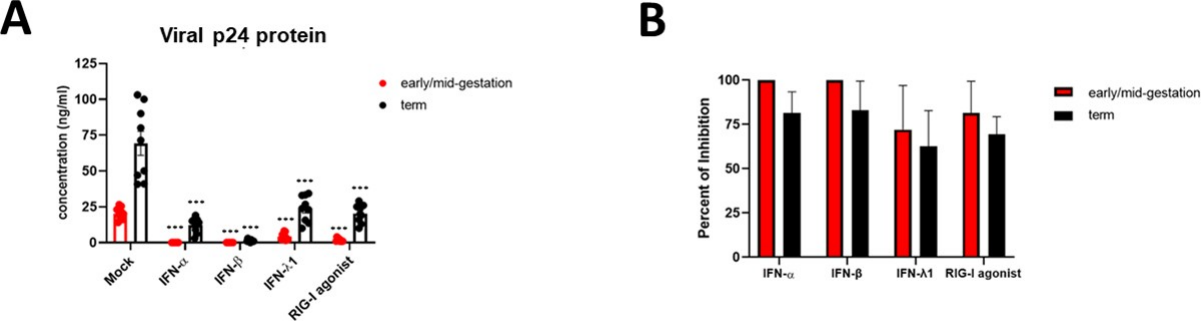
HIV infection is blocked by type I and type III signaling across gestation. *A*, Early/mid-gestation HCs (red markers) and term HCs (black markers) were infected with HIV-1_BaL_ alone or in the presence of IFN-α, IFN-β, IFN-λ1, RIG-I agonist, or mock-infected. HIV-1 replication was measured in the cell supernatants at 5 days post infection by HIV-1 p24 viral antigen enzyme-linked immunosorbent assay. Data shown represent individual donors (n = 10) analyzed by unpaired t-test analysis using the two-stage linear step-up procedure of Benjamini, Krieger and Yekutieli. ***p < 0.001 indicates significance between early/mid-gestation HCs compared to term HCs. *B*, Graphical representation of the percent inhibition of HIV-1 replication from *A*.

Given our findings that HIV infection of early/mid-gestation HCs induces an antiviral state, and the importance of the type I IFN signaling in the restriction of HIV infection, we next determined the ability of innate immune signaling to restrict HIV replication within placental macrophages. Here we pretreated HCs with IFNα, IFNβ, and IFNλ1 prior to infection and replenished the IFN-treated media 16hpi. In addition, to trigger RLR signaling, HCs were transfected with a highly specific RIG-I agonist prior to infection [54]. p24 levels were detected in supernatant 5dpi. Compared to untreated cells, we observed a drastic and significant reduction in viral replication in all cultures pretreated with type I and type III IFN or transfected with the RIG-I agonist (Fig 7A). Early/mid-gestation HCs demonstrated the greatest antiviral response to type I IFNs with 100% inhibition of viral replication following treatment (Fig 7A and 7B). In term HCs, IFNα and IFNβ also significantly reduced viral replication by approximately 80%. Similarly, IFNλ1 and treatment with the RIG-I agonist significantly reduced viral burden in early/mid-gestation and term HCs, albeit at a reduced potency when compared to treatment with the type I IFNs. *In sum*, these data demonstrate that type I and type III signaling can elicit a potent block to HIV replication in HCs at the maternal-fetal interface.

### Differential levels of STAT protein activation in term HCs may contribute to observed increases in HIV replication

**Fig 8:**
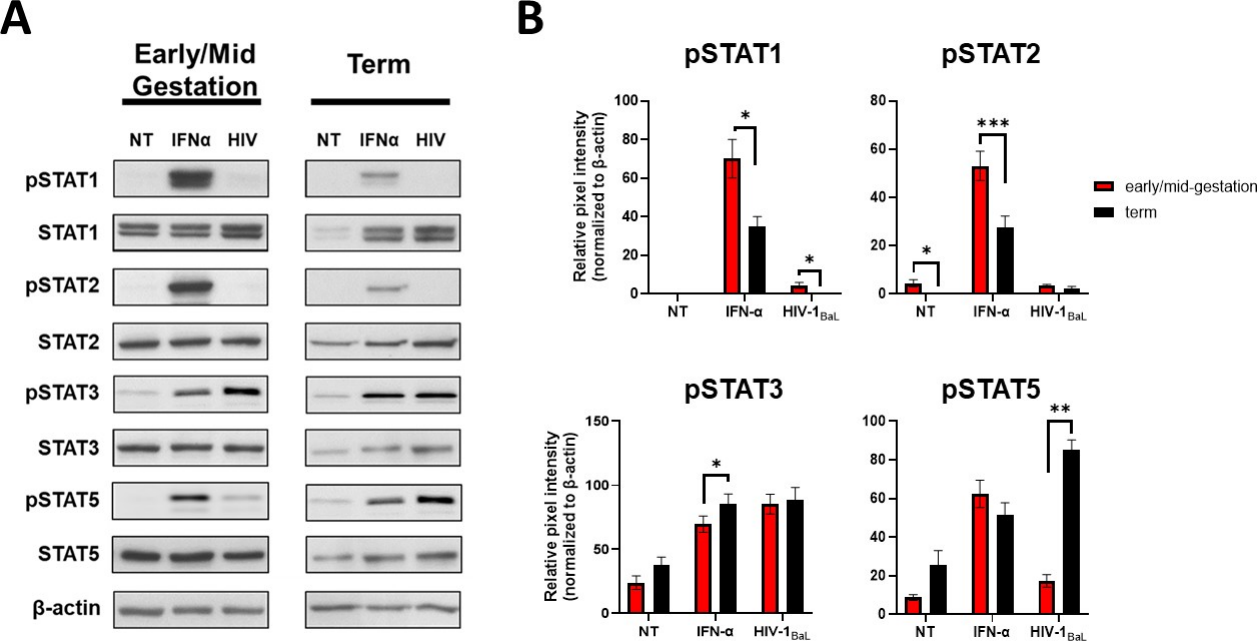
Differential Levels of STAT Protein Activation in Term HCs may contribute to observed Increases in HIV Replication. *A*, Hofbauer cells were infected with HIV-1_BaL_, treated with IFN-α (100 IU/mL), or left untreated (NT) for 72 hours. Lysates were prepared and subjected to sodium dodecyl sulfate–polyacrylamide gel electrophoresis. The gel was blotted and the indicated proteins were detected by immunoblotting with indicated antibodies. *B*, The pixel intensities for pSTAT1, pSTAT2, pSTAT3, and pSTAT5 of the untreated, IFN-α–treated and HIV-infected Hofbauer cells were quantified using the Odyssey Imaging system and normalized t the β-actin signal. Data shown are expressed as the mean ± standard error of biological triplicates from 10 individual donors analyzed by paired t-test analysis using the two-stage linear step-up procedure of Benjamini, Krieger and Yekutieli. *** p< 0.0001, **p < .001, and *p < .05 indicate significance between early/mid-gestation HCs compared and term HCs.

Engagement of type I IFNs to the IFN receptor complex leads to activation of STAT proteins by tyrosine phosphorylation. These complexes translocate to the nucleus and bind IFN-stimulated response elements (ISREs) in DNA to activate the transcription of hundreds of ISGs, which mediate antiviral responses [55, 56]. This signaling axis is critical for restricting most RNA viral infections including HIV. Our data shows that term HCs are limited in generating type I IFN responses, which may be responsible for observed increases in HIV infection and transmission during late gestation. However, we show significant upregulation of the STAT mRNA following HIV infection. To determine whether this increase in mRNA transcription translates to protein expression, we analyzed total protein levels and protein phosphorylation of STAT1, STAT2, STAT3, and STAT5 in HCs isolated at various gestational ages. We show that untreated and uninfected early/mid-gestation HCs express higher levels of total STAT1, STAT2, STAT3, and STAT5 compared to term HCs (Fig 8A). Our data also shows that early/mid-gestation cells express low levels of pSTAT2 at a basal level, while both early/mid-gestation and term HCs displayed constitutive levels of activated STAT3 and STAT5. Following IFNα treatment, STAT1 and STAT2 were also phosphorylated at a significantly greater level in early/mid-gestation HCs. In comparison, IFN-induced STAT3 exhibited higher levels of activation in term HC, compared to cells isolated from early/mid-gestation.

Interestingly, HIV-infection minimally induced the phosphorylation of STAT1 and STAT2 in both early/mid-gestation and term HCs. However, STAT3 and STAT5 phosphorylation was evident following HIV infection (Fig 8B). While the levels of STAT3 phosphorylation were similar among the HC subsets, STAT5 phosphorylation was drastically and significantly elevated in term HCs infected with HIV. Taken together, these data suggest that gestational age differentially impacts STAT protein expression and activation, and that robust activation of STAT5 following HIV infection of term cells may play a role in viral dissemination.

## Discussion

Very little information is known about how viruses establish infection in the placenta and developing fetus. Reports have suggested that gestational age plays a major role in the transmission of specific viruses; however, there are no mechanistic studies to support these observations. In particular, *in utero* HIV transmission has most often been documented in the third trimester. With an estimated 160,000 new HIV infections occurring worldwide in infants and children (most by MTCT), there is a pressing need to understand the host and viral factors that regulate vertical transmission. We sought to fill this knowledge gap by identifying the impact of gestation on viral susceptibility and antiviral signaling in placental macrophages (HCs). Here, we report that robust innate immune responses at the placenta during early gestation may offset vertical transmission of HIV *in utero*. We also demonstrate that term placental macrophages are limited in their antiviral capacity possibly due to restricted type I IFN signaling. Defining mechanisms and timing of vertical transmission are critical to understanding development and administration of specific vaccines and antiretrovirals to prevent new infections in HIV-exposed infants, globally.

Macrophages are a heterogeneous immune cell population and actively participate in a myriad of biological processes, including maintaining tissue homeostasis, viral sensing, and cell autonomous antiviral immune responses. These cells are characterized by a high level of plasticity, whereby their phenotype and function are strictly regulated by the local micro-environment [57]. The placental milieu is dynamic over the course of gestation and can be characterized by alternating pro- and anti-inflammatory states associated with fertilization, implantation, maintenance, preparation for labor, and parturition [58–61]. As such, the balance of macrophage polarization (M1 [pro-inflammatory] and M2 [anti-inflammatory]) at the maternal-fetal interface has emerged as vital in sustaining a healthy pregnancy. In normal pregnancy, the M1/M2 equilibrium is a continuum, with M1 predominating during implantation and early gestation, followed by a shift towards a tolerogenic M2 phenotype in the second and third trimesters, and a return to M1 at the start of parturition [62–67]. Recently, our group undertook a comprehensive *ex vivo* classification of human placental macrophages (HCs) throughout gestation [5]. We demonstrated that activated HCs were abundant in early pregnancy and noted molecular signatures, specific to inflammatory phenotypes early in gestation compared to term. In addition, the polarity and plasticity of HCs in response to cytokines and innate immune signals was differentially regulated across gestation [5]. These findings suggest that HCs recognize and respond to viral pathogens temporally at the various stages of pregnancy. Here, we extended these studies to show that HCs isolated from term placentae are more permissive to HIV infection than early/mid-gestation HCs. Although term HCs expressed lower frequencies of CCR5 on their surface, they secreted significantly lower levels of IFN-α and IL-10 and lacked productive type I IFN signaling following HIV-1 exposure. Alternatively, the lower viral burden in early/mid-gestation HCs may be associated with increased cellular immune activation, elevated levels of IL-10, along with robust RLR and type I IFN signaling. Thus, the capacity of HCs to support productive HIV-1 infection appears to be dependent on gestation and the pregnancy stage-specific microenvironment, which impacts the phenotype and immune responses in HCs.

Although CCR5 is a major co-receptor for the entry of HIV M-tropic strains into macrophages, a higher percentage of CCR5-expressing cells in early/mid-gestation did not equate with increased *in vitro* HIV replication. This biphasic effect is intriguing and might be due to CCR5 competition with inhibitory chemokines MIP-1α, MIP-1β, and RANTES, which are induced by HIV-1 replication. Previous studies have demonstrated that such competition might occur in the immediate vicinity of chemokine-secreting cells although the global concentrations obtained in culture supernatant does not reach inhibitory levels [68, 69]. In addition, HIV-infection induces IL-10 secretion in early gestation HCs. The inhibitory effect of IL-10 on HIV replication has been previously reported [70–72], where IL-10 did not decrease susceptibility to infection, but exhibited antiviral effect by limiting virus production. Similar heterogeneity has been noted in alveolar and vaginal macrophages, which are susceptible to HIV-1, whereas intestinal macrophages are typically resistant [73, 74]. HIV resistance in intestinal macrophages may be the result of high levels of IL-10 and TGF-β, which down-regulate expression of CCR5 and CXCR4, as well as innate signaling mediators [74, 75]. In earlier studies, we have demonstrated that HCs limit HIV-1 replication by the induction of IL-10 and TGF-β [28]; here we extend those studies to show that secreted immunoregulatory cytokines are significantly upregulated in HCs isolated early in gestation. Collectively, these findings suggest that functional polarization throughout gestation may be an important regulator of susceptibility to HIV-1 infection and efficiency of viral replication in placental macrophages.

The placenta is a robust immune organ that has the capability to respond to pathogens via PRRs. One particular PRR family, the RIG-I-like receptors (RLRs), respond to RNA viral infections, including HIV, signal downstream to produce type I IFNs, and upregulate IFN-stimulated genes (ISGs). At the maternal-fetal interface, type I IFNs (IFN-α and IFN-β) regulate inflammation, contribute towards fetal immunity, and are implicated in protection against viruses [76–78]. Type I IFNs are the first line of defense against viral infections and play a prominent role in control of HIV replication [79–81]. The production of type I IFNs is the hallmark of effective antiviral immunity, yet interestingly, many viruses can antagonize the IFN pathway to enhance infection and replication. Similar to type I, type III IFNs (IFN-λ1–4), which are secreted by placental trophoblasts, can mount antiviral responses and induce ISGs [82–85]. Here, we demonstrate that early/mid-gestation and term HCs constitutively secrete substantial levels of IFN-α in culture. This constitutive, baseline expression of type I IFNs in tissue has been shown to contribute to the modulation of the local immune responses and protection against viral infection [86]. During pregnancy, high levels of IFN-α may protect the fetus from acquiring intrauterine infections, such as herpes simplex virus [77]. Here we noted that early/mid-gestation HCs constitutively secrete significantly elevated levels of IFN-α and in response to HIV-1 infection, compared term HCs, underscoring that the placenta in early gestation may be less conducive to viral infection and dissemination. Along with IFN-α, others have noted that loss of IFN-β signaling in the placenta leads to uncontrolled viral replication, fetal infection, and maternal mortality [87, 88], and that IFN-λ secretion by trophoblasts confers protection against ZIKV infection [89, 90]. These studies suggest a critical role for type I and III IFNs as important mediators of antiviral signaling at the placenta. Indeed, we show that HCs across gestation, with or without HIV-infection, exhibited robust induction of type I and III IFN mRNA; however, we failed to detect IFN-β and IFN-λ1 protein in any of the supernatants. This finding is consistent with previous observations in ZIKV-infected HCs, where we did not observe IFN-β in the supernatants despite robust induction of transcripts [91, 92]. These results indicate that HCs may have an intrinsic deficiency in translation and synthesis of IFN-β. However, given the important role of IFN-α, -β, and -λ in protection against pathogens at the maternal-fetal interface, limiting viral infection via the production of IFNs is likely a coordinated effort between HCs, trophoblasts, and other resident maternal and fetal immune cells.

Type I IFNs invoke a potent antiviral state in the cell following stimulation of two IFN receptor subunits to activate the Janus kinase (JAK) family of cytoplasmic tyrosine kinases, JAK1, and tyrosine kinase 2 (TYK2). Phosphorylated JAKs activate the signal transducers and activator of transcription factors (STAT), STAT1, STAT2, STAT3, and STAT5 [93–96]. STAT4 and STAT6 can also be activated by type I IFNs, but activation of these proteins is restricted to certain cell types, such as endothelial cells [97, 98]. Similar to type I IFN, type III IFNs have been shown to activate the STAT proteins and IFN regulatory factor 9 (IRF9) [99]. These complexes translocate to the nucleus and bind IFN-stimulated response elements (ISREs) in DNA to activate the transcription of hundreds of ISGs, which mediate antiviral responses [55, 56]. We demonstrate that early/mid-gestation HCs respond more robustly to IFN treatment, compared to term HCs. Type I IFN-treated cultures upregulated IFN and ISG transcripts in HCs isolated from early gestation associated with low levels of HIV-1 replication. We also noted that HIV-1 infection induces a prominent antiviral state in early/mid-gestation HCs, characterized by transcription and secretion of IFN-α and significantly elevated transcription of the RLRs, STAT1, STAT5, ISG15, OAS1, IFIT1, IFIT2, IFIT3, and Viperin. This data confirms that early/mid-gestation HCs are primed and equipped to respond robustly through viral-induced IFN secretion, which may offset vertical transmission during early pregnancy. In comparison, IFN treatment and HIV infection of term HCs minimally triggered type I IFN signaling. We noted that transcription of the STAT1, STAT2, STAT3 and STAT5 was upregulated following HIV infection of term HCs. In fact, the increase observed in STAT2 and STAT3 was significantly greater than that observed in early/mid-gestation HCs. However, when we evaluated whether STAT transcription correlated with STAT protein levels and activation, we found that HIV-infected term HCs did not phosphorylate STAT2 and there was no significant difference in STAT3 phosphorylation. STAT5 phosphorylation was significantly upregulated at term, compared to early/mid gestation. The importance of STAT5 activity in during viral infection is largely unknown, however recent observations have identified key molecular links between STAT5 activity and the induction of HIV replication. Studies have demonstrated that overexpression of STAT5 increased virus production in unstimulated primary T cells–both the number of p24+ cells and their level of p24 production–suggesting that STAT5 promotes a permissive state for HIV infection [100]. In addition, several STAT5-activating cytokines were able to exert upregulatory effects on HIV replication in mononuclear phagocytes [101] suggested a potential role in controlling viral expression. Furthermore, STAT5 has been shown to bind directly to one or more putative DNA binding sites present in the U3 region of the HIV long terminal repeats (LTR), which could potentially lead to triggering or enhancing viral transcription [100, 102]. In this regard, STAT5 could become a promising antiviral drug candidate [46]. Viperin was the only antiviral effector that displayed substantial upregulation in term HCs, in response to HIV infection. Viperin, an ISG, is induced through the type I IFN pathway. However, recent studies have indicated that Viperin can also be upregulated independently of IFN, through an IRF1 or IRF3 mechanism, which can be activated by viral factors or by the peroxisomal MAVS signaling pathway [103, 104]. During pregnancy, IRF1 is an important regulator of the inflammatory response during human labor [105], and further studies are warranted to examine the role of Viperin and the IRFs in regulating HIV infection during pregnancy and parturition.

This study had two important limitations. First, the number of donors used in this study was restricted due to the difficulty in acquiring these specimens and the complexities of isolating the primary cells from fresh tissue. Although the overall numbers are relatively small, statistical differences were identified between cells isolated at different time points in gestation and between infected and mock-infected groups, indicating sample size was sufficient. Another limitation is that the placental macrophages were isolated from tissue and placed into single cell culture, which may not fully represent responses seen in an intact placenta. Placental macrophages are the most abundant immune cell in the placenta and play a key role in the synthesis of mediators involved in establishment and maintenance of pregnancy, fetal development including tissue modeling and maintaining healthy tissue homeostasis, parturition, local immune ontogeny, and maternal-fetal tolerance [106, 107]. In addition, these cells are a major target for HIV [21, 23, 28, 30, 32, 108]. However, the complex milieu, including the changing architecture of placental villi throughout gestation, are potential variables that cannot be fully recapitulated *in vitro*. It is likely that MTCT of HIV is likely influenced by a variety of factors, such as maternal immunity, placental integrity, trophoblast regulation, fetal cell responses, along with HC regulation. Further research is necessary to recreate more complex and physiologically meaningful placental models *in vitro*. Nevertheless, our data, expands the understanding of HIV susceptibility and transmission throughout pregnancy and highlights the potential of placental macrophages and innate immune signaling to limit HIV transmission during early gestation.

To summarize, our study provides mechanistic insights to support previous clinical observations that *in utero* HIV transmission likely occurs in the third trimester. We show that gestational age plays a significant role in the ability of HCs to generate robust antiviral responses, which are necessary to prevent or reduce viral burden. Specifically, we show that viral recognition and robust antiviral immune responses in placental cells during early gestation may prevent *in utero* HIV infection and that diminished antiviral responses observed in term HCs may promote viral transmission. Our studies also identify potential deficiencies in IFN-β translation throughout gestation and increases in total and phosphorylated STAT5 at term; both of which may be important host factors involved in viral transmission at the maternal-fetal interface. These findings are important in defining the mechanisms and specific timing of vertical transmission, which may contribute to the development of specific vaccines and antiviral therapies.

## Materials and methods

### Ethics statement

Early gestation human placentae were obtained from a free-standing clinic in GA from consented donors who elected to terminate pregnancies prior to 21 weeks and 6 days of gestation. Human term placentae (>37 weeks gestation) were collected from hepatitis B, HIV-1 seronegative women (>18 years of age) immediately after elective caesarean section without labor from Emory Midtown Hospital, Atlanta, GA. This study was approved by the Emory University Institutional Review Board (IRB 000217715). Written informed consent was acquired from all donors before sample collection. Samples were de-identified before primary HC isolation.

### Placental dissection and Hofbauer isolation

HCs were isolated from membrane-free villous placenta as previously described [28]. Briefly, the tissue was thoroughly washed and mechanically dispersed in Hank’s balanced salt solution (HBSS) to minimize peripheral blood contamination. The minced tissue was re-suspended in complete medium containing 10% Trypsin/EDTA (Sigma-Aldrich, St. Louis, MO, USA) for 1 hour, followed by resuspension in media containing 1 mg/ml collagenase A (Worthington Biochemical, Lakewood, NJ, USA) and 0.2 mg/ml of DNAse I (Sigma-Aldrich) and incubated in a shaking water bath at 37°C for 1 hour. The digested tissue was washed with PBS and passed through gauze and a 70 μm cell strainer (BD-Falcon Biosciences, Lexington, TN, USA). The mononuclear cell population was isolated by density gradient centrifugation on Histopaque-1077 (Sigma-Aldrich). CD14^+^ Magnetic Cell Sorting was performed using anti-CD14 magnetic beads (Miltenyi Biotech, Bergisch Gladbach, Germany) as recommended by the manufacturer. On average, the purity was >95%. After isolation, HCs were cultured in complete RPMI medium consisting of 1x RPMI (Corning Cellgro, Corning, NY, USA), 10% FBS (Optima, Atlanta Biologics), 2mM L-glutamine (Corning), 1mM sodium pyruvate (Corning), 1x Non-essential Amino Acids (Corning), 1x antibiotics (penicillin, streptomycin, amphotericin B; Corning) at 37°C and 5% CO2. HCs were treated with the following as indicated, following resuspension per the manufacturer’s instructions: 100IU/mL IFN-α A/D (Novus, Centennial, CO, USA), 100ng/mL IFN-β (Peprotech, Cranbury, NJ, USA), 100ng/mL IL-29 (Peprotech), 5’ppp-dsRNA (Invivogen, San Diego, CA, USA), and control for 5’ppp-dsRNA (Invivogen).

### Viral infection of Hofbauer cells

HIV-1 infection of HCs were performed as previously described [28, 108, 109]. 1.0 × 10^5^ cells/well in a 96-well plate (Corning) were infected at a multiplicity of infection (MOI) of 0.1 overnight at 37°C with the HIV-1 BaL strain (HIV-1_BaL_). This isolate was obtained through the NIH AIDS Reagent Program, Division of AIDS, NIAID, NIH: HIV-1Ba-L contributed by Dr. Suzanne Gartner, Dr. Mikulas Popovic and Dr. Robert Gallo [110]. Cells were then washed with PBS to remove unabsorbed virus and replenished with complete media. To monitor HIV production, cell supernatants were collected 5 days post-infection. Viral replication was detected by p24 released into the supernatant by enzyme-linked immunosorbent assay (ELISA) (Advanced BioScience Laboratories, Rockville, MD, USA). The HIV-1_BaL_ strain is R5-trophic and was isolated from infant lung tissue [110].

### Flow cytometry

Cells were washed with PBS and gently detached using 0.5 mM EDTA in PBS. Then, HCs (5 × 10^5^ per sample) were blocked for 10 minutes on ice with 0.25μl/sample Human TruStain FcX (BioLegend, San Diego, CA, USA) in FACS buffer (1x PBS, 0.1% BSA, 1mM EDTA) and live/dead stained for 10 minutes on ice with Calcein Violet 450AM (eBiosciences, San Diego, CA, USA). HCs were stained for surface markers (S1 Table) for 20 minutes on ice using 0.25μl/sample of the following anti-human antibodies in FACS buffer: CD14-APC [M5E2], CCR5-PE [3A9], DCSIGN-PE-Cy7 [9E9A8], CXCR4-BV605 [12G5], CD80-PE [2D10.4], CD86-FITC [2331], CD163-BV605 [GHI/61], and HLA-DR-PerCP-Cy5.5 [G46-6] (BioLegend and BD Biosciences, Franklin Lakes, NJ, USA). Stained cells were analyzed 48 hours post-infection on a BD LSR II flow cytometer driven by the DiVA software package (BD) following calibration using 6-peak Rainbow Calibration Particles (BioLegend). Analysis of the acquired data was performed using FlowJo software (Tree Star, Ashland, OR, USA). Compensation values were calculated using UltraComp eBeads (Life Technologies). Gating strategy is included (S1 Fig).

### RNA isolation and quantitative RT-PCR

Messenger RNA (mRNA) was extracted using the RNAeasy kit (Qiagen). The complementary DNA was transcribed using QuantiTect RT kit (Qiagen). HC gene expression was quantified by qRT-PCR using QuantiTect SYBR Green PCR Kits (Qiagen) with specific primers for host and integrated viral genes (S2 Table). Delta cycle threshold (ΔCt) values from the calibrator and experimental groups were measured by subtracting Ct values from target vs the housekeeping transcript, β-actin. Gene expression data are represented as ΔCt values or as fold change relative to paired, time-matched, mock-infected controls (gene expression normalized to β-actin − ΔΔCt method).

### Luminex

Cytokine and chemokine concentrations (S3 Table) in the supernatants of mock, HIV-infected, and/or IFN-treated HCs (500,000 cells per condition) and accompanying controls were assessed in duplicate using the Human Cytokine Magnetic 25-Plex Panel (Pub. No. MAN0003646; Invitrogen) per the manufacturers’ instructions. Plates were read on a Luminex 100 Analyzer and concentrations were determined by comparison to a 7-point standard curve.

### ELISA

IFN-λ1 and IFN-β concentrations in the supernatants of mock, HIV-infected, and/or IFN-treated HCs (500,000 cells per condition) and accompanying controls were assessed in duplicate using the Human IL-29/IFN-λ1 DuoSet ELISA Kit (DY7246; R&D Systems, Minneapolis, MN, USA) and the Human IFN-β ELISA Kit (41410; PBL, Piscataway, NY, USA) according to the manufacturers’ instructions. Plates were read on a SpectraMax 250 plate reader and concentrations were determined by comparison to a standard curve.

### Immunoblotting

Cells were lysed in radio-immunoprecipitation assay buffer (ThermoFisher) with protease inhibitors (Roche, Basel, Switzerland). Samples were subjected to denaturing sodium dodecyl sulfate–polyacrylamide gel electrophoresis. Gels were blotted on nitrocellulose membranes (GE Healthcare). After blocking with buffer (Li-Cor; Lincoln, NE, USA), the membrane was incubated with the following primary antibodies overnight at 4°C (S4 Table): mouse anti-STAT1 (ab155933), mouse anti-pSTAT1 (ab29045), mouse anti-STAT2 (sc-1668), rabbit anti-pSTAT2 (ab53132), mouse anti-STAT3 (ab119352), rabbit anti-pSTAT3 (ab76315), mouse anti-STAT5 (sc-74442), rabbit anti-pSTAT5 (ab32364), and mouse anti-β-Actin (ab8226) [Abcam, Cambridge, UK; Santa Cruz Biotechnology, Santa Cruz, CA, USA]. Next, the membrane was incubated with the appropriate IRDye goat anti-mouse and IRDye goat anti-rabbit secondary antibodies (Li-Cor). Prior to western blot analysis with the Odyssey Infrared Imaging System (Li-Cor), the combined linear range of detection for the targets and β-actin were determined. All images were analyzed using the Odyssey Application Software within the pre-determined ranges.

### Statistical analysis

All figures are representative of at least 3 independent experiments and 10 individual donors. Data were analyzed using multiple one-sample t-tests comparing mock, infected, or treated values. Analyses were corrected for multiple comparisons by controlling the false discovery rate. Discovery was determined using the two-stage linear step-up procedure of Benjamini, Krieger and Yekutieli, with Q = 5%, without assuming consistent SD [111]. Data were analyzed by two-tailed t-test using the two-stage step-up method by Benjamini, Krieger and Yekutieli. Differences were defined as significant when p ≤ .05. All statistical analysis was performed using GraphPad Prism 9.1.1 software. In each of the main and supplemental figure legends, “n” represents the number of placental donors from which HCs were derived. Further experimental statistical details are described in the figure legends.

## Supporting information

S1 Fig Gating Strategy for Hofbauer Cells. (TIF)

S1 Table Description of antibodies used in flow cytometry experiments. (DOCX)

S2 Table Primer Sequences for qRT-PCR. (DOCX)

S3 Table Tabulation of cytokine multiplex and ELISA data (pg/ml). (DOCX)

S4 Table Description of antibodies used in western blot experiments. (DOCX)
