# Supplementary figures and images for: Robust innate immune responses at the placenta during early gestation may limit in utero HIV transmission
Source: PLoS Pathog. 2021 Aug 25;17(8):e1009860. doi: 10.1371/journal.ppat.1009860 (PMC8437274; doi:10.1371/journal.ppat.1009860)

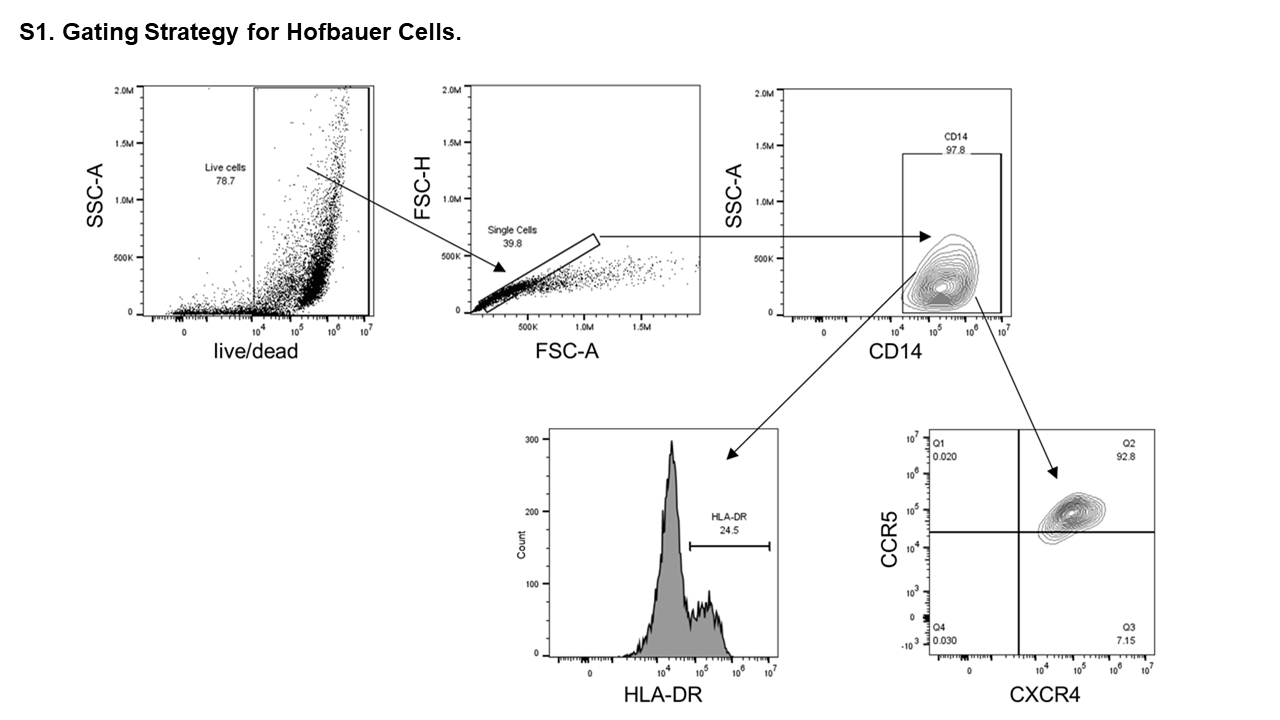

Supplement: S1 Fig — (TIF) [file ppat.1009860.s001.tif]
