# Supplementary material for: Robust innate immune responses at the placenta during early gestation may limit in utero HIV transmission
Source: PLoS Pathog. 2021 Aug 25;17(8):e1009860. doi: 10.1371/journal.ppat.1009860 (PMC8437274; doi:10.1371/journal.ppat.1009860)
Supplement: S1 Table — (DOCX) [file ppat.1009860.s002.docx]

| **Antibody** | **Fluorochrome** | **Clone** | **Provider** | **Catalog number** | **Isotype control** |
| --- | --- | --- | --- | --- | --- |
| CCR5 | PE | 3A9 | BD Biosciences, Franklin Lakes, NJ | 550632 | IgG2a, κ |
| CXCR4 | BV605 | 12G5 | BD Biosciences | 740418 | IgG2a, κ |
| DC-SIGN | PE-Cy7 | 9E9A8 | BioLegend, San Diego, CA | 330114 | IgG2a, κ |
| CD80 | PE | 2D10.4 | BD Biosciences | 566992 | IgG1, κ |
| CD86 | FITC | 2331 | BD Biosciences | 555657 | IgG1, κ |
| HLA-DR | PerCP-Cy^TM^5.5 | G46-6 | BD Biosciences | 560652 | IgG2a, κ |
| CD163 | BV605 | GHI/61 | BD Biosciences | 745091 | IgG1, κ |
| CD14 | APC | M5E2 | BD Biosciences | 555399 | IgG2a, κ |

**S1 Table. Description of antibodies used in flow cytometry experiments.**
