# Supplementary material for: Robust innate immune responses at the placenta during early gestation may limit in utero HIV transmission
Source: PLoS Pathog. 2021 Aug 25;17(8):e1009860. doi: 10.1371/journal.ppat.1009860 (PMC8437274; doi:10.1371/journal.ppat.1009860)
Supplement: S2 Table — (DOCX) [file ppat.1009860.s003.docx]

| **Gene** | **Forward Primer (5’- 3’)** | **Reverse Primer (5’- 3’)** |
| --- | --- | --- |
| ***β-ACTIN*** | GGCCCAGTCCTCCCCAAGTCCAC | GGTAAGCCCTGGCTGCCTCCACC |
| ***CCR5*** | AATAATTGCAGTAGCTCTAACAGG | TTGAGTCCGTGTCACAAGCCC |
| ***CXCR4*** | TGACTCCATGAAGGAACCCTG | CTTGGCCTCTGACTGTTGGTG |
| ***ENV*** | GGGGACCAGGGAGAGCATT | TGGGTCCCCTCCTGAGGA |
| ***GAG*** | ACATCAAGCAGCCATGCAAAT | ATCTGGCCTGGTGCAATAGG |
| ***IFNα*** | GACTCCATCTTGGCTGTGA | TGATTTCTGCTCTGACAACCT |
| ***IFNβ*** | GTCTCCTCCAAATTGCTCTC | ACAGGAGCTTCTGACACTGA |
| ***IFNλ1*** | GACGAGTACAGGCAGCTTCC | AGCATTGACCCTTAGGATCTTCTC |
| ***RIG-I*** | ATCCCAGTGTATGAACAGCAG | GCCTGTAACTCTATACCCATGTC |
| ***MDA5*** | GGCATGGAGAATAACTCATCAG | CTCTTCATCTGAATCACTTCCC |
| ***LGP2*** | ACGGGTGTATGCGCTTCAC | TTGCGGTCATCGAACAGGG |
| ***STAT1*** | CGGCTGATTTTCGGCACCT | CAGTAACGATGAGAGGACCCT |
| ***STAT2*** | CCCCCATGGCGCAGTGGGAAATGCTG | GGGGAATTCCTAGAAGTCAGAAGGCATC |
| ***STAT3*** | ACCAGCAGTATAGCCGCTTC | GCCACAATCCGGGCAATCT |
| ***STAT5*** | GCCGAGAAGCACCAGAAGACC | CGGCCAGCATCTCCTCCA |
| ***ISG15*** | TGACTGTGAGAGCAAGCAGC | CCCCAGCATCTTCACCTTTA |
| ***OAS1*** | CAAGCTCAAGAGCCTCATCC | TGGGCTGTGTTGAAATGTGT |
| ***IFIT1*** | AGAAGCAGGCAATCACAGAAAA | CTGAAACCGACCATAGTGGAAAT |
| ***IFIT2*** | GACACGGTTAAAGTGTGGAGG | TCCAGACGGTAGCTTGCTATT |
| ***IFIT3*** | AAAAGCCCAACAACCCAGAAT | CGTATTGGTTATCAGGACTCAGC |
| ***VIPERIN*** | CCAGTGCAACTACAAATGCGGC | CGGTCTTGAAGAAATGGCTCTCC |

**S2 Table.** **Primer Sequences for qRT-PCR**
