# Supplementary material for: Robust innate immune responses at the placenta during early gestation may limit in utero HIV transmission
Source: PLoS Pathog. 2021 Aug 25;17(8):e1009860. doi: 10.1371/journal.ppat.1009860 (PMC8437274; doi:10.1371/journal.ppat.1009860)
Supplement: S3 Table — (DOCX) [file ppat.1009860.s004.docx]

|  |  | Early/mid-gestation HCs | | | | Term HCs | | | |
| --- | --- | --- | --- | --- | --- | --- | --- | --- | --- |
|  | **LLOQ** | **Mock** | | **HIV-1_BaL_** | | **Mock** | | **HIV-1_BaL_** | |
|  |  | **Mean** | **SEM** | **Mean** | **SEM** | **Mean** | **SEM** | **Mean** | **SEM** |
| IL-1RA | 57 | 15480.1 | 1517 | 20124.1 | 1972.1 | 4544.2 | 919.3 | 4352.7 | 991.4 |
| IL-4 | 13 | 35.7 | 7.2 | 33.2 | 6 | 15.6 | 3.3 | 14.7 | 3.9 |
| IL-10 | 12 | 55.4 | 7.3 | 161.7 | 25.6 | 17.6 | 6.2 | 16.9 | 6.8 |
| MIP-1α (CCL3) | 18 | 54875.4 | 6437.7 | 148750.9 | 20216.5 | 8875.2 | 875 | 14003.4 | 1239.2 |
| MIP-1β (CCL4) | 25 | 91374.5 | 3432.7 | 112873.4 | 24692 | 18626.4 | 1116.8 | 24126.9 | 3617.3 |
| RANTES (CCL5) | 12 | 531.3 | 34 | 4819.1 | 418.5 | 87 | 6.3 | 104.5 | 3.8 |
| IL-1β | 12 | 82.6 | 53.9 | 193.1 | 98 | 42.7 | 10.8 | 91.7 | 23.3 |
| IL-6 | 7.02 | 4034.8 | 2685 | 30796.3 | 8719.4 | 1874.9 | 1021.8 | 7440 | 2433 |
| TNF-α | 5.75 | 9.9 | 3 | 11.4 | 4.4 | BD | - | BD | - |
| IFN-α | 10 | 87.2 | 8 | 210 | 26.6 | 44.1 | 4.6 | 50.4 | 5.9 |
| IFN-β | 50 | BD | - | BD | - | BD | - | BD | - |
| IFN-λ1 | 62.5 | BD | - | BD | - | BD | - | BD | - |

**S3 Table. Tabulation of cytokine multiplex and ELISA data (pg/ml).**
