# Supplementary material for: Robust innate immune responses at the placenta during early gestation may limit in utero HIV transmission
Source: PLoS Pathog. 2021 Aug 25;17(8):e1009860. doi: 10.1371/journal.ppat.1009860 (PMC8437274; doi:10.1371/journal.ppat.1009860)
Supplement: S4 Table — (DOCX) [file ppat.1009860.s005.docx]

| **Antibody** | **Host** | **Clone** | **Provider** | **Catalog number / RRID** | **Dilution** |
| --- | --- | --- | --- | --- | --- |
| STAT1 | Mouse | 15H3 | Abcam, Cambridge, UK | ab155933 | 1:1000 |
| pSTAT1 | Mouse | M135 | Abcam | ab29045 | 1:1000 |
| STAT2 | Mouse | A-7 | Santa Cruz Biotechnology, Santa Cruz, CA | sc-1668 | 1:500 |
| pSTAT2 | Rabbit | Y690 | Abcam | ab53132 | 1:500 |
| STAT3 | Mouse | 9D8 | Abcam | ab119352 | 1:5000 |
| pSTAT3 | Rabbit | EP2147Y | Abcam | ab76315 | 1:2000 |
| STAT5 | Mouse | A-9 | Santa Cruz | sc-74442 | 1:500 |
| pSTAT5 | Rabbit | E208 | Abcam | ab32364 | 1:1000 |
| β-Actin | Mouse | 8226 | Abcam | ab8226 | 1:1000 |

**S4 Table. Description of antibodies used in western blot experiments.**
